# Supplementary material for: Oxidoreductase gene fabG contributes to fungal development, cell wall integrity, and virulence in Aspergillus fumigatus
Source: Microbiol Spectr. 2025 Nov 13;14(1):e02092-25. doi: 10.1128/spectrum.02092-25 (PMC12772249; doi:10.1128/spectrum.02092-25)
Supplement: Supplemental Material — Tables S1 and S2; Fig. S1 and S2. [file spectrum.02092-25-s0001.docx]

**Table S1 Primers used for the study of deletion and complementary strains**

| Primer | Nucleotide Sequence(5’ to 3’) | Purpose |
| --- | --- | --- |
| P1 | AGGTACGGGCCTGAGAATCA | Amplify sequence  upstream of *fabG* |
| P2 | TAGTTCTGTTACCGAGCCGGTGTTGCTGTTTTTGGATGAAGCTA |  |
| P3 | GCTCTGAACGATATGCTCCAACATGCGCCCTTTGTATGATTTGTATA | Amplify sequence  downstream of *fabG* |
| P4 | AGCTAATGAGGACGGCACAGA |  |
| P5 | CATGTAGTGTTGAAGGACGGCAC | Fusion of upstream, downstream, and *PyrG* |
| P6 | CGAGTTATGAGTGAAAGTGGGAGG |  |
| PyrG-F | CCGGCTCGGTAACAGAACTACCGCAGACAATGCTCTCTATC | Amplify sequence of *PyrG* |
| PyrG-R | GTTGGAGCATATCGTTCAGAGCAATACCGTTACACATTTCCA |  |
| Aim-yz-F | CTCTGGGATATCAACAGCAGCAA | Amplify sequence of  *fabG* (for verification) |
| Aim-yz-R | GTTAACTTCATCACGAAGTAGGGTA |  |
| PyrG-yz-F | CTCGGTTGAGTACGCGCGGAAGTA | Amplify sequence of *PyrG* (for verification) |
| PyrG-yz-R | AGATGAGGAAGTTGTGCTTTGTC |  |
| Aim-Re-F | TCGAGGGGGGGCCCGAGTTTCTGAAGCTGAGCTTCAG | Amplify sequence of  *fabG* (for complementation) |
| Aim-Re-R | CTCGCCACGTTCGCCTTACAAGCTTAGGGGATAGCCCTACCCC |  |
| Hph-yz-F | ATCGTTATGTTTATCGGCACTTTG | Amplify sequence of *hph*(for verification) |
| Hph-yz-R | TGTTGGCGACCTCGTATTGG |  |

**Table S2 Primers used in study for RT-qPCR**

| Primer | Nucleotide Sequence(5’ to 3’) | Purpose |
| --- | --- | --- |
| *sod1*-q-F | TCCAAGCAGGACAAGCTTATTA | *sod1* |
| *sod1*-q-R | CCAGCGTTACCAGTCTTCTT |  |
| *catA*-q-F | GGAGCCAGGAGTTTCAAGATAA | *catA* |
| *catA*-q-R | CAGATGGAAGGTGTGGTAGAC |  |
| *mkk2*-q-F | CCAAGAGCCGGATTGATAGAT | *mkk2* |
| *mkk2*-q-R | AGTTATCGGACCAGCGAATAC |  |
| *chsA*-q-F | GGATGATGACAGGGCCACAA | *chsA* |
| *chsA*-q-R | ATCAAACGCCTCGGAACTGT |  |
| *chsB*-q-F | GAAACACACTTCACCCGCAC | *chsA* |
| *chsB*-q-R | AGTGCGTTCAGAGTCCTTGG |  |
| *chsC*-q-F | CGGATGTCGGGATACAGTGG | *chsC* |
| *chsC*-q-R | TCAACGCTGAGTACCGAACC |  |
| *chsG*-q-F | ATGTGCCACCAGTCGAGAAG | *chsG* |
| *chsG*-q-R | AGCAGAATTCGTGAAGCCGA |  |
| *fksA*-q-F | CACTTCTTTCCTTGCACGCC | *fksA* |
| *fksA*-q-R | ACGTTCTCTGGGTTCTGCTG |  |
| *gelA*-q-F | TGCGTCAGTACATCCGTAGC | *gelA* |
| *gelA*-q-R | GATCGCACCAGGAGTAGTCG |  |
| *gelB*-q-F | CACCAAGGACAAGGACCCT | *gelB* |
| *gelB*-q-R | CTGCGTTGAAGATGGATGCG |  |
| *rlmA*-q-F | CATCCCATGCCTCAACCAGT | *rlmA* |
| *rlmA*-q-R | CTTGTTCGGGCAAGAAAGGC |  |
| *csmB*-q-F | AGGAGGGCGGAGGATGGATG | *csmB* |
| *csmB*-q-R | CAAGTGCGGTGAAGGCTATGC |  |
| *agsA*-q-F | TCATTGGCCTCGCCCATTTC | *agsA* |
| *agsA*-q-R | TGACATAAGCCTGCTGCGTG |  |
| *agsC*-q-F | ACCCCTCAGATACTGGTGCC | *agsC* |
| *agsC*-q-R | CACCGTCCAACGAAGACGAG |  |
| *tubA*-F | TTCCCAACAACATCCAGACC | *tubA* |
| *tubA*-R | CGACGGAACATAGCAGTGAA |  |
| IL-1β -F | TATGAGCTGAAAGCTCTCCACCTC | IL-1β |
| IL-1β-R | GCCGTCTTTCATTACACAGGACA |  |
| TNF-α-F | AAATGGCCTCCCTCTCATCAGT | TNF-α |
| TNF-α-R | GGTGGTTTGCTACGACGTGG |  |
| IL-6-F | CTGCAAGAGACTTCCATCCAG | IL-6 |
| IL-6-R | AGTGGTATAGACAGGTCTGTTG |  |
| GAPDH-F | ACCCAGAAGACTGTGGATGGC | GAPDH |
| GAPDH-R | TCAGATCCACACGACGGACAT |  |


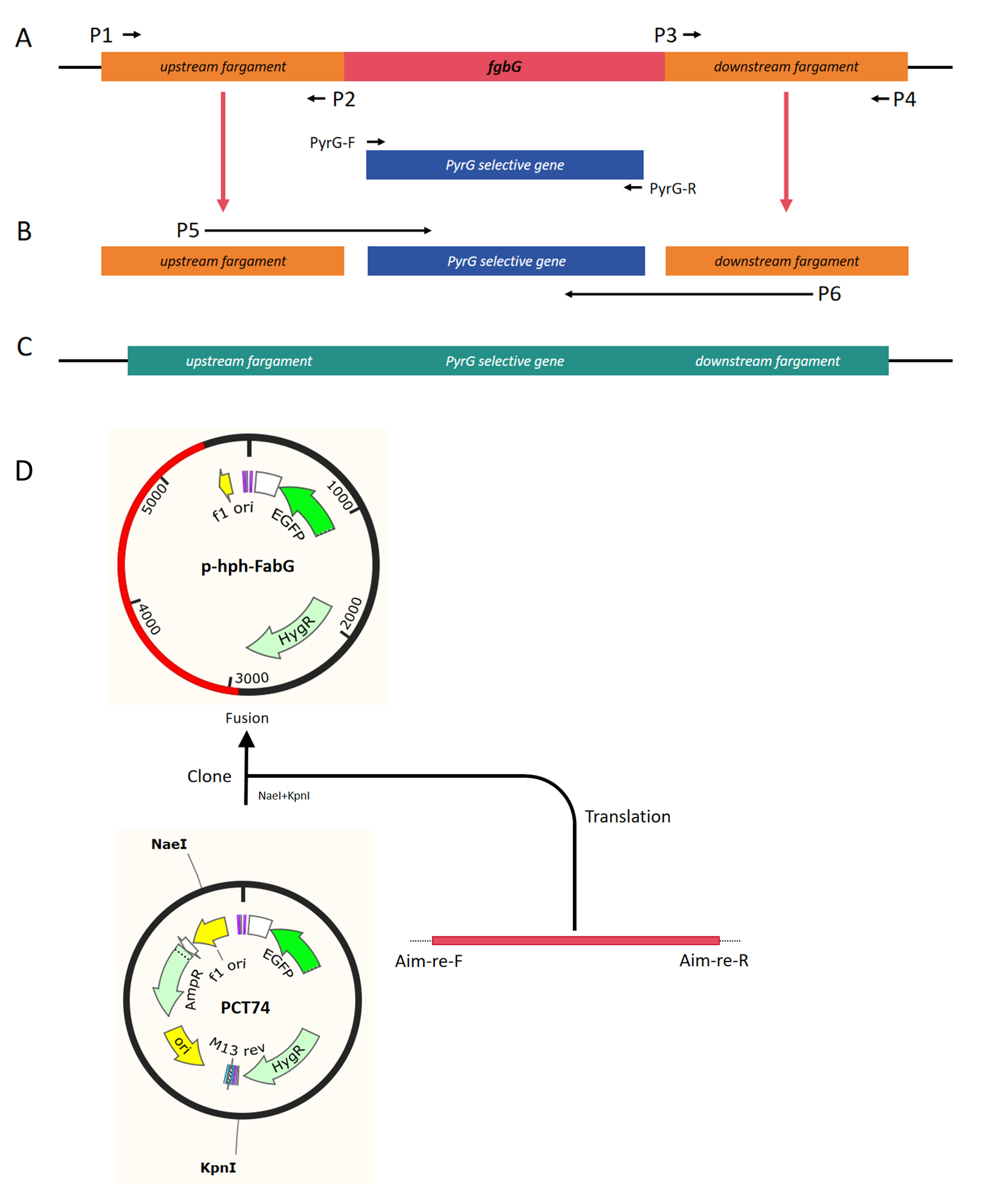


**Fig. S1 Construction of the *fabG* mutant and reconstituted strain.**

(A) Amplification of homologous fragments and selection marker. The upstream and downstream homologous regions flanking fabG were amplified using primer pairs P1+P2 and P3+P4, respectively. The *pyrG* auxotrophic marker was amplified with primers PyrG-F+ PyrG-R. (B) Fusion PCR assembly of the knockout cassette. The three fragments (upstream, *pyrG*, downstream) were fused via overlap PCR using primers P5+P6 to generate the *fabG* deletion construct. (C) Schematic of the *fabG* knockout cassette. The final construct contained the *pyrG* marker flanked by upstream and downstream homologous arms for targeted gene replacement.(D) Complementation of Δ*fabG* with the WT allele. To complement the Δ*fabG* mutant, the *fabG* allele was amplified using primers Aim-Re-F and Aim-Re-R. The pCT74 was then digested with NaeI and KpnI to excise the hygromycin resistance (HygR) cassette. The *fabG* fragment was subsequently ligated into the linearized pCT74 using Hieff Clone® Plus Multi One Step Cloning Kit(Yeasen, China), generating the complementation plasmid p-hph-fabG.


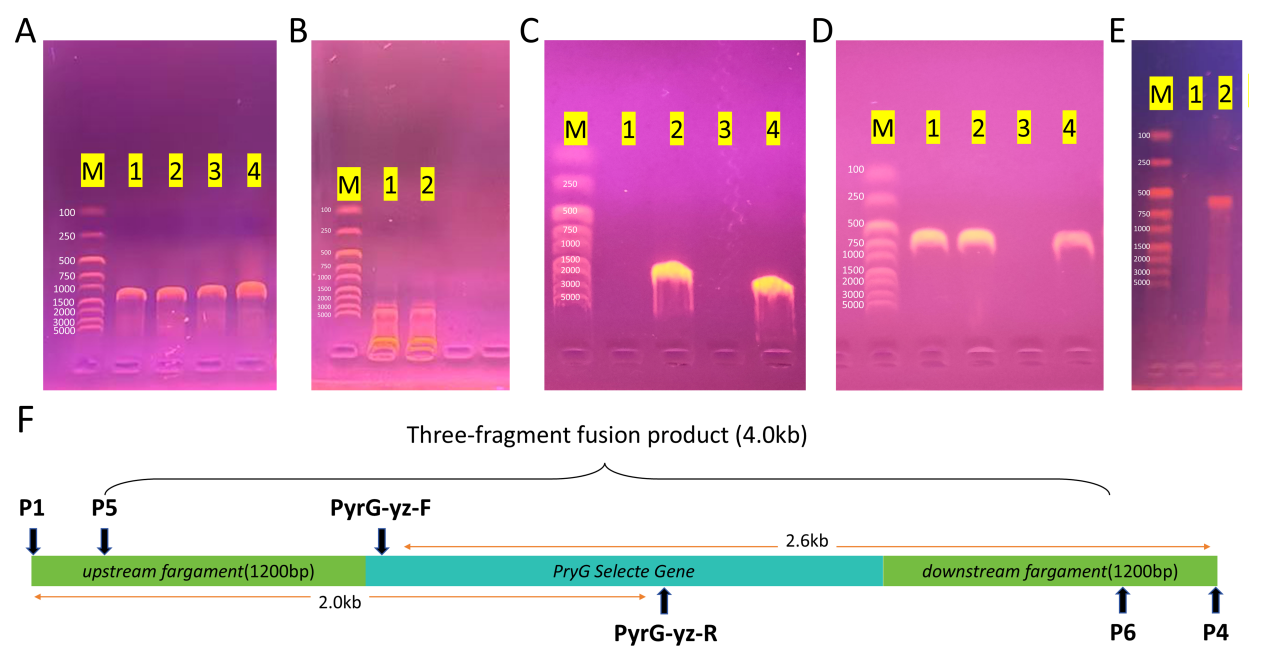


**Fig. S2 Verification of the construction process for knockout and complemented strains.**

(A) M, Marker; Lane 1 and 2, amplification of the ***pyrG*** gene; Lane 3 and 4, amplification of the upstream and downstream regions of the target gene.
(B) M, Marker; Lane 1 and 2, verification of the three-fragment fusion product.
(C) M, Marker; Lane 1 and 2, amplification of the **WT** and Δ*fabG* strain DNA sequences using **P1+PyrG-yz-R** primers, with clear bands present in the Δ*fabG*; Lane 3 and 4, amplification of the **WT** and Δ*fabG* strain DNA sequences using **P4+PyrG-yz-F** primers, with clear bands present in the Δ*fabG*.
(D) M, Marker; amplification of the target gene using **Aim-yz-F+Aim-yz-R** primers, Lane 1 to 4 correspond to **WT**, complemented plasmid p-hph-fabG, Δ*fabG*, and Δ*fabG::fabG^+^*, respectively.
(E) M, Marker; amplification of the ***hph*** gene using **Hph-yz-F+Hph-yz-R** primers, Lane 1 is **WT**, Lane 2 is the Δ*fabG::fabG^+^*.

(F) Figure illustrating the validation of the knockout strain.
